# Emerging Molecular and Biological Functions of MBD2, a Reader of DNA Methylation

**DOI:** 10.3389/fgene.2016.00093

**Published:** 2016-05-26

**Authors:** Kathleen H. Wood, Zhaolan Zhou

**Affiliations:** Department of Genetics, Perelman School of Medicine, University of Pennsylvania, PhiladelphiaPA, USA

**Keywords:** methyl-CpG binding domain protein, MBD2, MBD3, DNA methylation, nucleosome remodeling and histone deacetylation complex (NuRD), chromatin, mouse genetics, transcription regulation

## Abstract

DNA methylation is an epigenetic mark that is essential for many biological processes and is linked to diseases such as cancer. Methylation is usually associated with transcriptional silencing, but new research has challenged this model. Both transcriptional activation and repression have recently been found to be associated with DNA methylation in a context-specific manner. How DNA methylation patterns are interpreted into different functional output remains poorly understood. One mechanism involves the protein ‘readers’ of methylation, which includes the methyl-CpG binding domain (MBD) family of proteins. This review examines the molecular and biological functions of MBD2, which binds to CpG methylation and is an integral part of the nucleosome remodeling and histone deacetylation (NuRD) complex. MBD2 has been linked to immune system function and tumorigenesis, yet little is known about its functions *in vivo*. Recent studies have found the MBD2 protein is ubiquitously expressed, with relatively high levels in the lung, liver, and colon. *Mbd2* null mice surprisingly show relatively mild phenotypes compared to mice with loss of function of other MBD proteins. This evidence has previously been interpreted as functional redundancy between the MBD proteins. Here, we examine and contextualize research that suggests MBD2 has unique properties and functions among the MBD proteins. These functions translate to recently described roles in the development and differentiation of multiple cell lineages, including pluripotent stem cells and various cell types of the immune system, as well as in tumorigenesis. We also consider possible models for the dynamic interactions between MBD2 and NuRD in different tissues *in vivo*. The functions of MBD2 may have direct therapeutic implications for several areas of human disease, including autoimmune conditions and cancer, in addition to providing insights into the actions of NuRD and chromatin regulation.

## Introduction

### DNA Methylation and Its Readers

DNA methylation is a chemical epigenetic modification that is essential for mammalian viability and development. DNA methylation at CpG dinucleotides (mCG) has historically been associated with stable gene repression; however, recent advances have revealed a complex role for DNA methylation in regards to its dynamic turnover, cell type-specific distribution patterns, and effect on transcriptional regulation (Suzuki and Bird, 2008; Smith and Meissner, 2013). DNA methylation is also found outside of the CpG context (mCH), particularly in embryonic stem cells (ESCs) and brain tissues (Lister et al., 2009, 2013; Guo et al., 2014; Schultz et al., 2015). While mCG is the most abundant modification, mCH is detected at significantly lower frequencies compared to mCG in non-neuronal tissues. The oxidized form of methylcytosine, hydroxymethylation (hmC), is particularly enriched in ESCs and the brain (Lister et al., 2009, 2013; Guo et al., 2014; Schultz et al., 2015).

DNA methylation, particularly in the CpG context, is intimately linked to histone modifications, formation of heterochromatin, and transcription factor recruitment. Together these mechanisms comprise the chromatin state and direct gene expression programs. The genomic distributions of these modifications are developmentally dynamic and cell type-specific, but the mechanisms that direct the interpretation of these patterns to affect specific gene expression programs have yet to be fully determined. It has recently been found that DNA methylation affects transcriptional regulation differently depending on the genomic context. For example, mCG at promoters is associated with transcriptional repression, while the same mark in gene bodies is associated with high levels of gene transcription (Schübeler, 2015). It is critical to gain further understanding of the mechanisms of DNA methylation in transcriptional regulation because these findings will aid in our elucidation of other biological processes, including the differentiation of pluripotent cells, neuronal development and function, and tumorigenesis, among many others.

**FIGURE 1:**
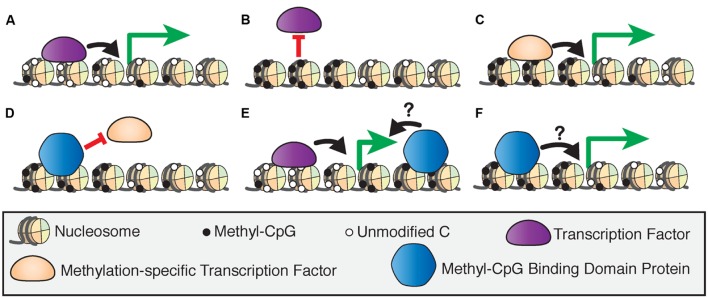
**Models of 5-methylcytosine affecting transcription factor recruitment.** **(A)** The binding of transcription factors at hypomethylated regulatory regions drives transcriptional activation. **(B)** Activating transcription factors are blocked from binding hypermethylated regulatory regions, inducing transcriptional silencing. **(C)** Methylation-specific transcription factors bind hypermethylated regulatory regions to activate transcription. **(D)** Methyl-CpG binding domain (MBD) proteins can bind hypermethylated regions and block transcription factor binding to induce transcriptional silencing. MBD proteins bind to actively transcribed genes at intragenic **(E)** or promoter **(F)** sites, but the effect of this binding on transcriptional regulation is unclear.

DNA methylation can directly influence transcription by affecting how and where protein factors bind to DNA. Transcription factors can be repelled by methylation at promoters which leads to gene silencing (Blattler and Farnham, 2013; Domcke et al., 2015), but can also bind to specific methylated sequences in association with active transcription (**Figures 1A–C**; Spruijt and Vermeulen, 2014). DNA methylation can also indirectly guide transcription through the specific binding of several classes of proteins referred to as ‘readers’ of CpG methylation, which can then recruit other chromatin-modifying proteins to DNA (Spruijt et al., 2013). Here, we focus on the methyl-CpG binding domain (MBD) family of ‘reader’ proteins (Hendrich and Bird, 1998).

The MBD proteins are a key component of epigenetic regulation as they act at the intersection of several critical mechanisms that affect transcriptional regulation. The MBD proteins may induce transcriptional silencing by blocking transcription or other protein factors from binding to DNA (**Figure 1D**), or by inducing chromatin remodeling through their binding partners (Nan et al., 1998). However, there is evidence that these proteins are also bound at actively transcribed genes in promoters or intragenic sites, although the effect of this binding on transcriptional regulation is not fully understood (Baubec et al., 2013; Spruijt et al., 2013; **Figures 1E,F**). This and other new evidence shows that the MBD proteins are dynamic readers of methylation beyond their originally proposed roles as transcriptional repressors, and therefore are essential to our understanding of epigenetic processes. Despite many biochemical and genetic studies on the MBD proteins, the precise functions of these proteins *in vivo* are yet to be fully investigated.

### The MBD Protein Family

**FIGURE 2:**
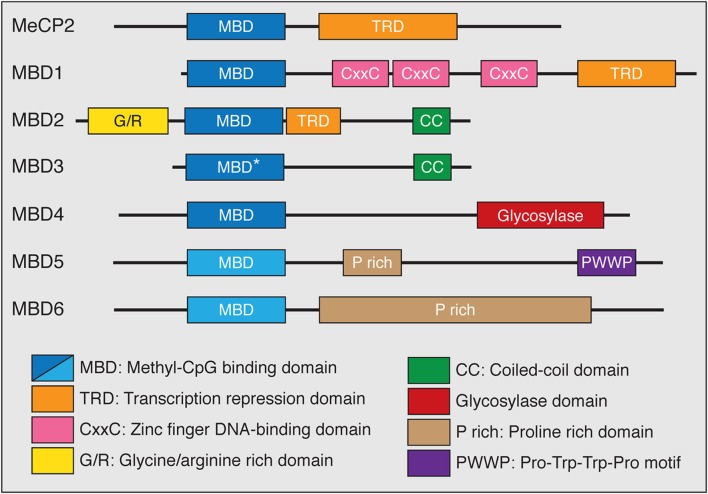
**The MBD family of proteins.** All MBD family proteins contain a highly conserved MBD (blue box) in addition to other functional domains. The MBD of MBD3 has a point mutation (^∗^) that abolishes methyl-CpG binding. MBD5 and MBD6 do not specifically bind methyl-CpG (lighter blue box). MeCP2, MBD1, and MBD2 contain C-terminal transcriptional repression domains (TRD) that interact with co-repressor protein complexes (orange box). MBD1 has CxxC-type zinc finger domains which mediate DNA binding. MBD2 has an N-terminal glycine/arginine rich domain (yellow box) and a C-terminal coiled-coil domain, which is also found in MBD3 (green box). The functions of MBD4 are mediated through a C-terminal glycosylase domain (red box). Both MBD5 and MBD6 contain proline rich domains (tan box) while MBD5 has a PWWP motif (purple box) that binds methylated histones.

The functions of the MBD family of proteins have been of great interest because these proteins have been genetically linked to disease in humans. The MBD family represents a group of proteins that generally act as mediators between methylation, primarily in the CpG context, and other chromatin and histone modifying protein complexes (Du et al., 2015). The MBD protein family consists of MeCP2 and MBD1-6 (**Figure 2**). Despite the name, not all members of this family bind to mCG with exclusivity, or at all. Instead the MBD proteins have distinct DNA-binding properties and other functional domains that may contribute to their respective functions. MeCP2, MBD1 and MBD2 bind to DNA in a mCG-density dependent manner via the MBD and associate with co-repressor and other protein complexes through their transcriptional repression domains (TRDs; Nan et al., 1998; Ng et al., 1999; Fujita et al., 2000; Feng and Zhang, 2001; Baubec et al., 2013). MBD1 can bind to unmodified cytosine through its CxxC-type zinc finger domains in addition to recognizing mCG through its MBD (Jørgensen et al., 2004). MBD3 has a point mutation in the MBD domain that abolishes its selective binding to mCG, and instead binds with low affinity to unmodified cytosine, mCG, and hmC (Hashimoto et al., 2012a; Spruijt et al., 2013). MBD4 binds to methylated DNA and has DNA glycosylase activity that is unique in the MBD family (Hashimoto et al., 2012b). The most recently described MBD proteins, MBD5 and MBD6, are localized at pericentric heterochromatin but do not specifically bind methylated DNA (Laget et al., 2010).

In this review, we examine and contextualize research on MBD2. This MBD protein is highly conserved, ubiquitously expressed, and interacts with the nucleosome remodeling and histone deacetylation (NuRD) complex (Hendrich and Bird, 1998; Hendrich et al., 2001; Hendrich and Tweedie, 2003; Wood et al., 2016). Surprisingly, *Mbd2* null mice show only mild phenotypes compared to mice with loss of function of other MBD proteins (Hendrich et al., 2001; Wood et al., 2016). One proposed explanation for this discrepancy is that some amount of functional redundancy exists among the MBD proteins (Baubec and Schübeler, 2014). However, biochemical and genetic evidence suggests that MBD2 has unique functions that have recently been shown to contribute to transcriptional regulation in pluripotent cells, immune lymphocytes, and in tumorigenesis. Here, we address the molecular functions of MBD2, roles for MBD2 in biological processes and human disease, and models of MBD2 interaction with the NuRD complex.

## MBD2 Molecular Functions

### MBD2 Gene and Protein Structure

**FIGURE 3:**
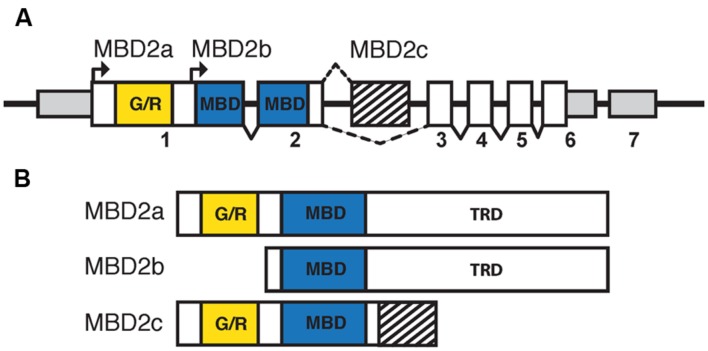
**The isoforms of MBD2 include different functional domains.** **(A)** The *Mbd2* transcript has seven exons, with non-coding regions (smaller gray box) in exons 1, 6, and 7. Coding regions are contained in exons 1–6 (white and filled boxes). The MBD (blue box) is split between exons 2 and 3 with the glycine/arginine rich region (yellow box) in exon 1. There are two translation start sites corresponding to MBD2a and MBD2b, respectively. MBD2c includes an alternatively spliced exon (striped box) between exons 2 and 3. **(B)** MBD2a utilizes the first translation start site and includes the G/R rich domain, MBD and C-terminal TRD. Translation of MBD2b excludes the N-terminal G/R rich domain. MBD2c includes the alternatively spliced exon and does not contain the C-terminal TRD.

Mammalian MBD2 was identified in a search for proteins containing the conserved MBD (Hendrich and Bird, 1998). Human and murine MBD2 and MBD3 have a highly similar genomic structure, likely indicating the occurrence of an ancestral gene duplication event (Hendrich and Tweedie, 2003). Murine MBD2 is encoded by six coding and one non-coding exons, with the MBD spanning exons 2 and 3, and has three isoforms: MBD2a, MBD2b, and MBD2c (also known as MBD2t; **Figure 3A**; Hendrich and Bird, 1998; Hendrich et al., 1999). The distinctions between the isoforms of MBD2 correspond to different functions and binding partners, and are therefore critical for the understanding of MBD2 function *in vivo*. All MBD2 isoforms contain the MBD, which binds specifically to mCG with no demonstrated affinity for unmodified cytosine, hmC, or mCH (Hashimoto et al., 2012a; Spruijt et al., 2013).

MBD2a and MBD2b arise from two alternative translation start sites and differ only in the inclusion of a GR-rich N-terminal domain in MBD2a (**Figure 3B**). Both isoforms contain the full MBD and C-terminal TRD, which is essential for MBD2 interactions with co-repressor protein complexes, including the NuRD complex (Boeke et al., 2000). The inclusion of the GR-rich domain in MBD2a may have important functional consequences, as post-translational methylation of this region affects interactions with DNA and NuRD (Tan and Nakielny, 2006). MBD2a protein consistently appears as a doublet in western blot analyses, suggesting that a fourth alternatively spliced, translated, or cleaved isoform may be present (Ng et al., 1999; Wood et al., 2016). The C-terminal TRD region of MBD2 includes two domains that interact with different members of the NuRD complex (Feng and Zhang, 2001; Gnanapragasam et al., 2011; Desai et al., 2015). The third isoform, MBD2c or MBD2t, utilizes an alternative third exon and produces a truncated protein that includes the N-terminal GR-rich domain and the MBD, without the C-terminal TRD (**Figure 3B**; Hendrich and Bird, 1998). This isoform is expressed exclusively in the testes and ESCs and does not interact with the NuRD complex, with important functional consequences particularly for pluripotent stem cells (Hendrich and Bird, 1998; Baubec et al., 2013; Lu et al., 2014).

### MBD2 and NuRD

**FIGURE 4:**
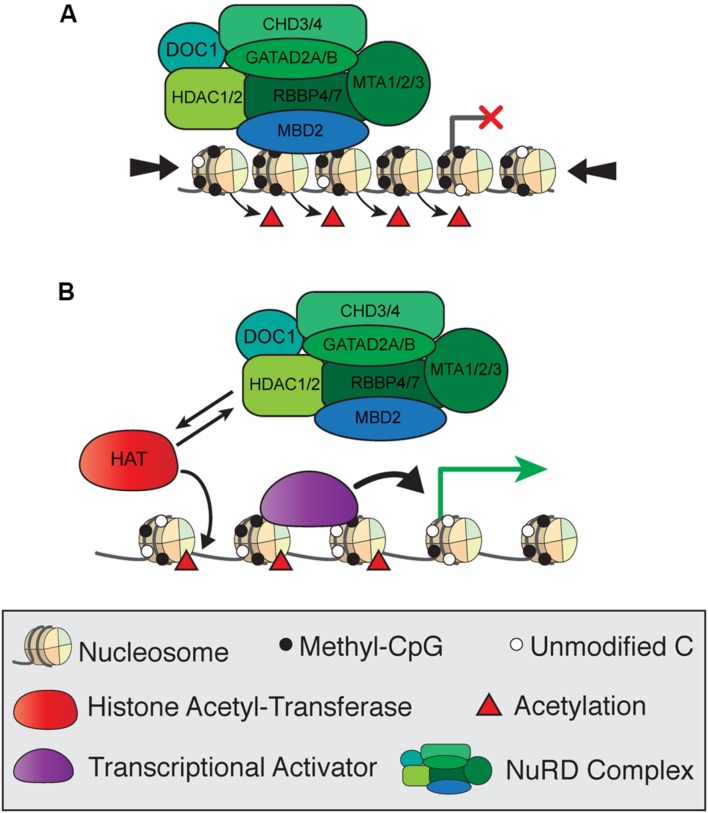
**Simplified model of transcriptional regulation by MBD2 and NuRD.** **(A)** MBD2 binds to mCG-dense regions as a component of the NuRD complex, which induces histone deacetylation and chromatin compaction (large arrows) leading to transcriptional silencing. **(B)** At transcriptionally active sites, MBD2/NuRD is replaced by histone acetyltransferases and activating transcription factors to induce histone acetylation and open, active chromatin.

The composition and functions of the NuRD complex, including MBD2, are conserved from mammals to other vertebrates and insects (Wade et al., 1999; Marhold et al., 2004). NuRD consists of the ATP-dependent remodeling enzymes CHD3/4, histone deacetylases HDAC1/2, histone chaperones RBBP4/7, and DNA binding proteins GATAD2A/B and MTA1/2/3 in addition to MBD2 and MBD3, and has both ATP-dependent nucleosome remodeling and histone deacetylase activity (Torchy et al., 2015). Early studies of MBD2 protein function focused on the transcriptional repressive activities of NuRD and were based on reporter assay in cultured cells. The first model of MBD2 function proposed that MBD2 recruits NuRD to methylated regions of the genome to induce histone deacetylase-dependent transcriptional silencing and chromatin compaction (**Figure 4A**; Ng et al., 1999). In this simplified model, reduced mCG density corresponds to less MBD2/NuRD binding activity, increased histone acetylation, and more open chromatin allowing for active transcription (**Figure 4B**). This mechanism was observed in numerous studies in cultured cells examining repression of methylated reporter constructs and endogenous methylated promoters of several genes, particularly those related to cancer (Ng et al., 1999; Feng and Zhang, 2001; Auriol et al., 2005; Chatagnon et al., 2009; Ramírez et al., 2012).

Despite the many studies of MBD2 as a transcriptional repressor, it has been challenging to identify specific methylated genes that are directly targeted for regulation by MBD2 when studying transcriptome changes on a genome-wide scale. By correlating MBD2 binding sites determined by chromatin immunoprecipitation (ChIP) with differentially expressed genes upon knockdown of MBD2 in heterologous cells, several studies support the model that loss of MBD2 primarily results in de-repression of lowly expressed genes (Günther et al., 2013; Devailly et al., 2015). However, both upregulation and downregulation of many genes occurs upon loss of MBD2 in these cell cultures (Günther et al., 2013; Stefanska et al., 2013). It was proposed that loss of MBD2 may affect NuRD recruitment, thus leading to alteration of gene transcription (Reynolds et al., 2013).

**FIGURE 5:**
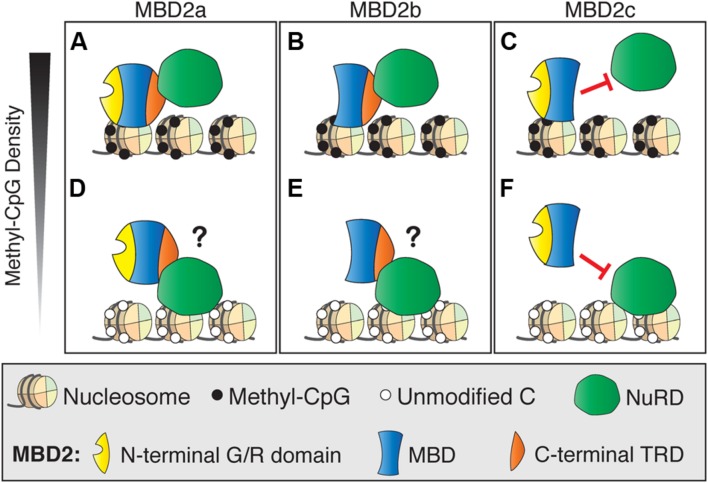
**Different DNA binding and NuRD interactions of MBD2 isoforms.** **(A)** MBD2a binds to methyl-CpG dense sites with the NuRD complex. **(B)** At unmethylated sites, MBD2 recruitment to DNA is dependent on interactions with NuRD through the C-terminal TRD. **(C)** Similarly to MBD2a, MBD2b binds to methyl-CpG dense sites with NuRD and may be recruited to unmethylated sites via interaction with NuRD **(D)**. MBD2c lacks the C-terminal TRD and does not interact with NuRD. Therefore, MBD2c may bind methylated sites without NuRD **(E)** and, in this model, would not be recruited to unmethylated sites by NuRD **(F)**.

One possible explanation for the subtle gene regulation by MBD2 is that the interactions between MBD2 and NuRD are more complicated than originally realized. This may be due to the presence of different isoforms of MBD2, of which only two interact with NuRD, and/or post-translational modifications of MBD2, which can also affect NuRD recruitment (Tan and Nakielny, 2006; Baubec et al., 2013). Recent findings are in opposition to the original model which proposed that MBD2 recruits NuRD to methylated sites (Ng et al., 1999). Surprisingly, NuRD shows less than expected co-occupancy with MBD2 at methylated genomic regions (**Figure 5A**). MBD2 is also found at unmethylated, actively transcribed genes and requires interaction with the NuRD complex for this localization. These findings suggest NuRD is recruiting MBD2 to unmethylated sites where it would otherwise not bind (**Figure 5B**). It is plausible that MBD2b, which has the C-terminal TRD, interacts with NuRD and DNA in a similar manner to MBD2a regardless of DNA methylation, but this has not been shown directly (**Figures 5C,D**). In contrast, MBD2c, which does not interact with NuRD due to the absence of the TRD, may still bind methylated sites without NuRD but is absent from unmethylated sites (**Figures 5E,F**). These findings support the view that MBD2, through its interactions with NuRD, may be involved in transcriptional activation as well as repression, and may be localized at unmethylated sites (Baubec et al., 2013).

The other critical factor that may be affecting MBD2 function is the role of MBD3 in complex with NuRD. MBD3 has repressive activity as part of the NuRD complex, despite not binding to mCG (Wade et al., 1999), but its exact functions within NuRD and the mechanisms and implications of NuRD assembly with either MBD2 or MBD3 are unknown. Several studies gave strong indication that MBD2 and MBD3 have distinct functions with NuRD *in vivo.* First, NuRD incorporates MBD2 or MBD3 into mutually exclusive complexes (Le Guezennec et al., 2006) and second, genetic knockout studies in mice show distinctly different phenotypes for each gene (Hendrich et al., 2001). It is also clear that MBD2/NuRD and MBD3/NuRD have distinct genome-wide distributions, reflecting their different DNA-binding properties, and different functions, particularly in ESCs (Kaji et al., 2006; Gu et al., 2011; Baubec et al., 2013; Günther et al., 2013; Lu et al., 2014). Together these findings suggest that transcriptional regulation by MBD2 is more dynamic and multifaceted than originally proposed, and this may help explain why direct targets of MBD2 have been difficult to identify. Further work is required to determine the dynamics of MBD2, MBD3, and NuRD recruitment to chromatin, and how MBD2 may function within and independently of NuRD.

### MBD2 and Other Protein Complex Interactions

**FIGURE 6:**
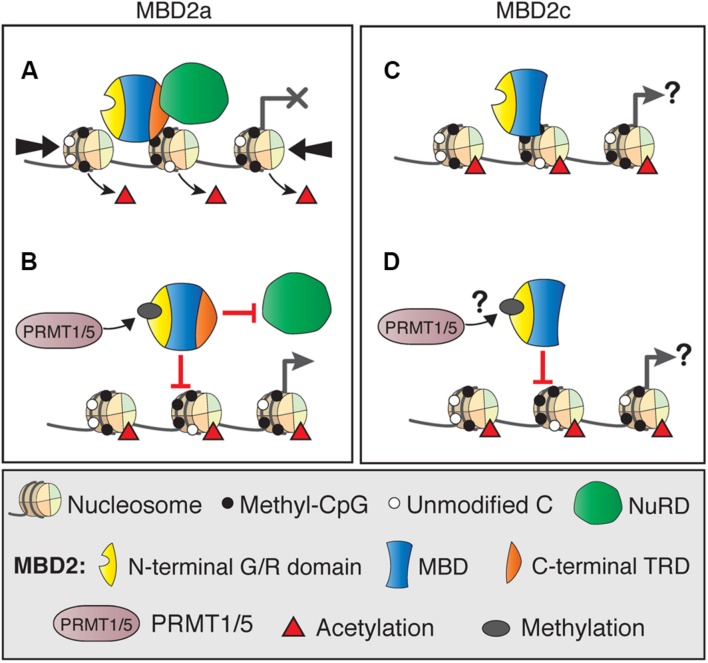
**DNA binding dynamics of MBD2 isoforms depend on post-translational modifications.** **(A)** MBD2a and NuRD binding to methylated sites results in nucleosome remodeling (thick arrows) and deacetylation (red triangles), producing transcriptional silencing. **(B)** Post-translational methylation of the N-terminal glycine/arginine rich region of MBD2a by PRMT1/5 reduces the affinity of MBD2a for NuRD and methylated DNA and may not produce transcriptional repression. **(C)** MBD2c does not interact with the NuRD complex but does bind methylated DNA. It is not fully understood how this binding may affect transcriptional regulation. **(D)** MBD2c has the N-terminal G/R rich domain, but it is not clear if it is also methylated by PRMT1/5 and if this modification would affect DNA binding similarly to MBD2a.

Although MBD2 is usually associated with the NuRD co-repressor complex, there is increasing evidence that MBD2 functions may rely on interactions with several other diverse protein complexes. Several of these interactions directly affect MBD2 binding to NuRD, and it is possible that they also mediate NuRD-independent functions. The most well-described example is the post-translational methylation of MBD2 by PRMT1 and PRMT5 (Le Guezennec et al., 2006; Tan and Nakielny, 2006). Methylation of the N-terminal RG-rich region of MBD2a (**Figures 6A,B**), and presumably MBD2c, although this has not been shown directly (**Figures 6C,D**), reduces the affinity of MBD2 for the NuRD complex and for methylated DNA. Importantly, these interaction may represent an MBD2-specific mechanism to regulate the formation or function of the NuRD complex, as MBD3/NuRD does not interact with the PRMT proteins (Le Guezennec et al., 2006). Methylation of histone H4 by PRMT1 or PRMT5 produces transcriptionally active or repressed chromatin, respectively (Nicholson et al., 2009). It is unknown if PRMT chromatin-modifying activity is affected by MBD2 interactions. For example, PRMT may be guided to methylate H4 where MBD2/NuRD is localized, or these functions may be independent of each other.

There is also surprising evidence that MBD2 is required for active transcription in several contexts, including at least two genes that are silenced by mCG (Wang et al., 2013; Weaver et al., 2014). Further studies in cultured cells showed that MBD2 can form a complex with TACC3 and the histone acetyltransferase pCAF to activate transcription (Angrisano et al., 2006). Additionally, MBD2a, but not MBD2b, reactivates transcription of unmethylated, cAMP-responsive genes through interactions with RNA helicase A, part of the CREB transcriptional coactivator complex (Fujita et al., 2003). An intriguing finding from both of these studies is that when MBD2 is associated with either of these protein complexes, it is not bound to histone deacetylases, which are key components of the NuRD complex. It is not clear if these interactions represent additional NuRD-independent functions of MBD2, or if they only serve to mediate MBD2-NuRD interactions.

Further work is necessary to resolve the many remaining questions surrounding the mechanisms of MBD2 function both in association with and independent of the NuRD complex. The majority of studies on MBD2 protein complex interactions have been performed in cell culture systems, and there is little information on these mechanisms in a relevant biological context. A mouse with biochemically tagged endogenous MBD2 that was recently described could be used to address these questions *in vivo* (Wood et al., 2016).

### Genome-Wide Binding Patterns and Transcriptional Regulation

The binding of MBD2 to methylated DNA depends on the presence of an intact MBD (Baubec et al., 2013). Several studies have examined MBD2 binding to specific loci such as the methylated regulatory regions of *BRCA1* or *Foxp3* (Auriol et al., 2005; Wang et al., 2013), but only a few attempts have been made to determine the genome-wide distribution of MBD2. These experiments are complicated by the fact that DNA methylation patterns are dynamic and cell-type specific (Schultz et al., 2015) and reliable antibodies to MBD2 are limited; therefore all ChIP data to date have been acquired using cultured cells or biochemical tagging approaches. These studies in HeLa cells (Chatagnon et al., 2011; Günther et al., 2013), mouse ESCs (Baubec et al., 2013), and breast cancer cell lines (Menafra et al., 2014; Devailly et al., 2015) all showed that MBD2 is broadly associated with densely methylated genomic regions, with no detectable sequence specificity. These studies found MBD2 is enriched at transcription start sites, promoters, and exons that coincide with methylated CGIs. Highly methylated sites that have low mCG density, such as most repetitive regions and low-CpG promoters, introns and intergenic regions, show low or no enrichment for MBD2 (Baubec et al., 2013; Günther et al., 2013; Menafra et al., 2014).

Surprisingly, MBD2 was also detected at certain unmethylated sites including intermediate- and high-CpG promoters that correlate with the presence of activating histone marks. It is currently unclear what proportion of cellular MBD2 is localized at these sites, as opposed to methylation-dense regions of the genome. These distribution patterns may in fact be different localization of different MBD2 isoforms. The MBD2c isoform, which lacks the C-terminal region that interacts with NuRD, was lost from these regions but retained at methylated sites, which suggests that MBD2 localization to unmethylated sites is NuRD-dependent. MBD2 binding to unmethylated promoters is also associated with tissue-specific regulatory regions and low levels of gene expression (Baubec et al., 2013; Günther et al., 2013; Menafra et al., 2014). In contrast, MBD3 binding, similar to NuRD, is not correlated with methylation and instead is enriched at transcriptionally active, open chromatin regions (Baubec et al., 2013; Günther et al., 2013). Despite evidence from several studies that MBD2/NuRD and MBD3/NuRD are localized at active chromatin, biochemical evidence shows that NuRD is repelled by the activating histone mark H3K4me3 (Eberl et al., 2013). Therefore, the mechanisms of MBD2/NuRD recruitment and distribution on chromatin remain to be further investigated. Additionally, the dynamics and biological consequences of NuRD formation with either MBD2 or MBD3 are unclear, particularly *in vivo* in the cellular context. It is also essential to recognize that to date all studies of MBD2 genome-wide localization have been performed in cultured cells. A mouse expressing a tagged allele of MBD2 (Wood et al., 2016) could be utilized to perform ChIP-seq in various cell types to explore these questions in an *in vivo* setting.

## MBD2 Biological Functions

### Neuronal Functions of MBD2 in Context of Related MBD Proteins

Epigenetic mechanisms in the brain are distinct because DNA methylation changes dynamically throughout development and in learning and memory processes (Lister et al., 2013; Heyward and Sweatt, 2015). The highly complex network of diverse neuronal subtypes each have distinct epigenomic and transcriptional profiles (Mo et al., 2015), further suggesting that epigenetic regulation is essential for the maintenance and function of the brain. Most MBD proteins are associated with neuronal functions in both humans and mice. The most well-studied example is *MECP2*, which when mutated, deleted, or duplicated causes the neurodevelopmental disorder Rett syndrome (Amir et al., 1999). Loss of MBD5 is the causative factor in 2q23.1 microdeletion syndrome, characterized by intellectual disability, behavioral problems and seizures (Talkowski et al., 2011). Mutations in the other MBD proteins have been correlatively linked to autism spectrum disorder (ASD; Li et al., 2005; Cukier et al., 2010, 2012). Mouse models with deletion of *Mecp2* or *Mbd5* closely recapitulate the symptoms of the respective human disorders, reflecting the conserved functions of these proteins (Guy et al., 2001; Camarena et al., 2014). Mutations in *Mbd1* in mice affect adult neurogenesis and result in behavioral changes considered to be representative of ASDs in humans (Zhao et al., 2003; Allan et al., 2008). A *Mbd3* brain-specific conditional null mouse has defects in cortical thickness and neuronal differentiation and is neonatal lethal (Knock et al., 2015).

With this evidence, it is surprising that *Mbd2* null mice show only mild phenotypes, including deficits in pup nurturing and nesting behaviors, hypoactivity, and low body weight (Hendrich et al., 2001; Wood et al., 2016). There are several possible interpretations of these findings. First, MBD2 may be dispensable for brain function, unlike most other MBD proteins that are clearly required. Alternatively, it is possible that loss of MBD2 does impair neuronal functions, but in too small a population of cells to produce robust changes in behavior. For example, *Mbd2* null mice have changes in the proliferation and differentiation of olfactory receptor neurons without notable changes in olfactory-dependent behaviors (Macdonald et al., 2010; Wood et al., 2016).

**FIGURE 7:**
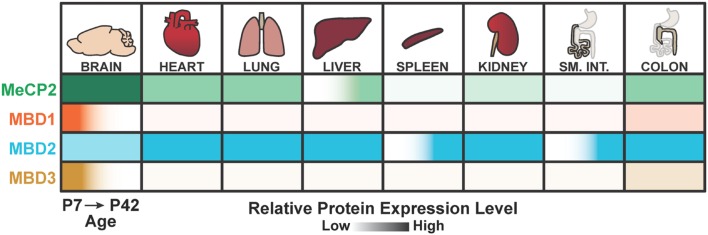
**Summary of MeCP2, MBD1, MBD2, and MBD3 spatiotemporal expression patterns.** MBD2 is expressed throughout the body with lower relative expression in the brain compared to other MBD proteins at postnatal day 7 (P7) and postnatal day 42 (P42). MBD2 is also up regulated temporally in the adult spleen and small intestine. In contrast, MeCP2 is most highly expressed in the brain. MBD1 and MBD3 are highly expressed in the brain at early ages, but down regulated in the adult brain. Adapted from Wood et al. (2016).

Another model commonly put forth is that MBD2 functions in the brain are at least partially redundant with other MBD proteins, which may be compensating for the loss of MBD2 at the cellular or systemic level. The most likely candidates for compensation are MeCP2, which is abundant in the brain but has quite different molecular functions to MBD2 (Du et al., 2015), or MBD3, as an alternate member of the NuRD complex (Le Guezennec et al., 2006). However, recent investigations into the spatiotemporal expression patterns of the MBD proteins showed that MBD3 is most highly and selectively expressed in the brain at younger ages in mice, and lowly expressed in the adult brain. In contrast, MBD2 shows consistent expression during development in multiple tissues, particularly the lung, liver, and colon (**Figure 7**; Wood et al., 2016). This evidence suggests that, at least in the adult brain, MeCP2 may be the dominant methylation ‘reader’ protein because MBD1 and MBD3 are lowly expressed while MBD2 appears to be dispensable for brain function. However, this scenario would also necessarily indicate that any NuRD-independent functions specific to MBD2, but not MBD3, are also dispensable for brain function.

A recent study revealed a function for MBD2 in the hippocampus that may underlie the maternal nurturing phenotypes in the *Mbd2* null mouse (Hendrich et al., 2001; Weaver et al., 2014). This study found that MBD2 expression is upregulated in response to maternal licking and grooming and was required for upregulation of the glucocorticoid receptor (GR) gene in the hippocampus (Weaver et al., 2014). Hippocampal GR signaling affects the regulation of stress and maternal nurturing behaviors through the hypothalamic–pituitary–adrenal (HPA) axis (Liu et al., 1997). According to this model, a female *Mbd2* null mouse born to a heterozygous mother would have an attenuated GR expression response to maternal behavior, leading to long-term epigenetic changes at the GR locus. These epigenetic changes could in turn affect this *Mbd2* null female mouse’s own maternal nurturing behaviors, as has been observed in female rats whose dams were exposed to environmental stress (Liu et al., 1997; Francis et al., 1999; Weaver et al., 2004). GR signaling has widespread, systemic effects related to stress, metabolism and inflammation (Kadmiel and Cidlowski, 2013), but it is unknown if loss of MBD2 affects these signaling mechanisms outside the hippocampus.

### Isoform-Specific Roles in Pluripotent Cells

The maintenance, proliferation, and differentiation of pluripotent cells are highly dependent on epigenetic mechanisms (Meissner, 2010). It is clear that MBD3 has an essential role in pluripotent ESCs because *Mbd3* null mice are early embryonic lethal (Hendrich et al., 2001). In contrast, mice with loss of any other MBD protein are viable, albeit with a range of phenotypic severity (Du et al., 2015). Recent work has revealed an important role for MBD2 in pluripotent cells. MBD2 is more lowly expressed in ESCs compared to MBD3 (Lu et al., 2014) and was initially thought to repress reprogramming of somatic to induced pluripotent stem (iPS) cells (Lee et al., 2013). However, further efforts found that differentially spliced isoforms of MBD2 actually have a role in both repression and promotion of reprogramming to pluripotency in human pluripotent stem cells (hPSCs; Lu et al., 2014). *MBD2a*, the longest isoform that includes both the N-terminal GR-rich domain and C-terminal TRD, is specifically enriched in differentiated fibroblasts (DFs) while alternatively spliced *MBD2c*, which lacks the TRD, is enriched in hPSCs. Overexpression of *MBD2a* in hPSCs disrupts pluripotency, presumably by mediating NuRD targeting to the *OCT4* and *NANOG* promoter regions and down-regulating their expression. In contrast, *MBD2c* is also bound at these promoters but does not interact with NuRD. Overexpression of *MBD2c* together with other reprogramming factors in DFs actually enhances the reprogramming efficiency. The authors conclude that *MBD2a* and *MBD2c* mediate the balance between proliferation and differentiation of hPSCs (Lu et al., 2014).

It is unclear why *Mbd2* constitutive null mice are viable and fertile when there is a role for MBD2 and NuRD in pluripotent cells (Hendrich et al., 2001; Reynolds et al., 2012; Lu et al., 2014). Similarly to the brain, it is possible that MBD3 may be sufficient to mediate NuRD-related functions in the absence of MBD2 during embryogenesis. However, a genetic interaction between *Mbd2* and *Mbd3* argues that MBD3 cannot fully compensate for the loss of MBD2 (Hendrich et al., 2001) and further studies are needed to resolve this question.

### Emerging Roles for MBD2 in Immunity

There is strong evidence that DNA methylation and other epigenetic mechanisms are essential in hematopoiesis and differentiation of myeloid and lymphoid cell lineages (Álvarez-Errico et al., 2014; Shih et al., 2014). NuRD, MBD2, and MBD3 have roles in multiple cell populations (Dege and Hagman, 2014). For example, the core NuRD component CHD4 is required for the maintenance and differentiation of hematopoietic stem cells (Yoshida et al., 2008). MBD2 is the only MBD protein among MeCP2, MBD1, and MBD3 that is expressed highly in the spleen, a site for induction of both innate and adaptive immune responses. Intriguingly, MBD2 is upregulated temporally in the adult spleen and not detectable at younger ages (**Figure 7**; Wood et al., 2016).

A specific role for MBD2 in early hematopoietic stem cells and other progenitor cells has not been described. *Mbd2* null mice have unaltered lymphoid organs and major lymphocyte subsets, suggesting that MBD2 may not be required at the early stages of hematopoiesis (Hutchins et al., 2002). In B cells, the NuRD complex interacts with various transcription factors to mediate temporal changes in development and differentiation (Gao et al., 2009; Musselman et al., 2012). Transcriptional regulation of the B-cell specific *Cd79a* gene involves MBD2-dependent CHD4 recruitment, but whether B cells are broadly affected in *Mbd2* null mice is unknown (Ramírez et al., 2012).

The functions of NuRD and MBD2 in several T cell populations have been studied more extensively. Loss of MBD2 has been linked to changes in proliferation or maturation of multiple T cell populations. These changes may arise from altered expression of several critical factors, some of which are controlled by differentially methylated regulatory regions. For example, loss of MBD2 or loss of NuRD components CHD4 or MTA2 skews CD4+ T cell polarization toward Th2 populations, with implications for pathogen resistance (Hutchins et al., 2002, 2005; Lu et al., 2008; Hosokawa et al., 2013). One study proposed that these changes may occur because MBD2/NuRD regulates expression of the Th2 cytokine locus, which is demethylated during Th2 cell differentiation (Aoki et al., 2009). Interestingly, MBD2 also indirectly affects CD4+ T cell maturation by regulating gene expression programs in dendritic cells, which are required to direct T helper cell maturation (Cook et al., 2015). The maturation of CD8+ T cell populations into effector and memory cells after acute viral infection is also directly affected by loss of MBD2, consistent with MBD2 regulating expression of surface markers and cytokines (Kersh, 2006). Finally, several studies found MBD2 regulates the expression of the master Treg cell transcription factor Foxp3 (Lal et al., 2009; Wang et al., 2013). MBD2 binds to a Treg-specific demethylation region (TSDR) upstream of *Foxp3* that becomes demethylated in thymus-derived natural Tregs. MBD2 promotes the TET2-mediated demethylation of the TSDR in Treg cells. Consequently, *Mbd2* null mice show decreased Treg numbers and impaired Treg suppressive function in addition to retaining methylation at the TSDR (Wang et al., 2013).

Disruptions to the NuRD complex are detrimental to immunity, as evidenced by a study that showed mice with loss of MTA2 develop a severe lupus-like autoimmune disease (Lu et al., 2008). Although loss of MBD2 results in reduced numbers of Treg cells, *Mbd2* null mice surprisingly do not develop autoimmunity, perhaps because their T effector cells are less responsive to stimulation and more susceptible to Treg suppression (Wang et al., 2013). In fact, loss of MBD2 is protective against experimental autoimmune encephalomyelitis, a model of T cell mediated autoimmunity and demyelinating diseases of the central nervous system (Zhong et al., 2014). In human patients, increased levels of MBD2 and global demethylation in CD4+ T cells has been observed in several autoimmune disorders, including systemic lupus erythematosus (Balada et al., 2007; Liu et al., 2011), systemic sclerosis, dermatomyositis (Lei et al., 2009) and *MBD2* was determined to be a susceptibility locus for psoriasis (Tsoi et al., 2012). These human and mouse studies point to MBD2/NuRD being an essential regulator of immune function with therapeutic potential. However, considerable effort is required to fully understand the complexities of MBD2 function in immunity at the cellular and systemic levels.

### Implications for the Role of MBD2 in Cancer

Epigenetic factors have been studied extensively in regards to cancer initiation, progression, and treatments (Baylin and Jones, 2011). The NuRD complex may affect tumorigenesis by modifying expression or activities of transcription factors linked to cancer, silencing hypermethylated tumor suppressor genes, and/or maintaining genomic stability (Lai and Wade, 2011). Many studies have attempted to link loss of MBD2 or MBD3 to significantly increased cancer predisposition in human patients, but evidence for this is limited. Therefore, the focus of studies on MBD2 and MBD3 in cancer has shifted to their potential as therapeutic targets. However, concerns have been raised regarding the feasibility of directly targeting these proteins and possible off-target effects (Parry and Clarke, 2011).

MBD2 has been studied particularly in the context of colorectal cancer, but questions remain as to the specific role of MBD2 in tumor initiation or progression. Interestingly, the colon is the only tissue besides the brain to show notable co-expression of MeCP2, MBD1, MBD2, and MBD3, suggesting the reading of mCG may be particularly important in this tissue (**Figure 7**; Wood et al., 2016). MBD2 is required for regulation of gene expression in the gastrointestinal tract, as *Mbd2* null mice show altered spatial expression of several genes in the small intestine and colon (Berger et al., 2007). Loss of MBD2 is protective against tumorigenesis specifically in *Apc^Min/+^* mice, a mouse model of sporadic colorectal tumorigenesis (Sansom et al., 2003). The *Apc^Min/+^*mouse develops tumors due to significantly upregulated Wnt signaling (Sansom, 2004), which is downregulated in the absence of MBD2 (Phesse et al., 2008).

While these results suggest targeting MBD2 may have therapeutic potential for colorectal cancer, further investigations show that the downregulation of Wnt signaling may be attributed to general disruption of chromatin regulation rather than MBD2-specific functions. First, *Mbd2* null mice in a wild-type background do not show significant changes in intestinal histology or Wnt signaling (Phesse et al., 2008). More importantly, similar downregulation of Wnt signaling and reduced tumorigenesis occurs when perturbing several other chromatin binding or modifying factors, including the DNMTs (Cai et al., 2014), the methylation-binding protein Kaiso (Prokhortchouk et al., 2006), and the chromatin remodeling factor Brg1 (Holik et al., 2014). In contrast, MBD3 is more likely to have a direct function in the gastrointestinal tract. Loss of MBD3 specifically in the gut results in increased tumorigenesis induced by inflammation through the upregulation of targets of the AP-1 transcription factor (Aguilera et al., 2011). It is currently unknown if these pathways are also affected by loss of MBD2.

Loss of MBD2 has complex effects on gene expression with both upregulation and downregulation of many genes, which differentially affects tumorigenesis in various mouse cancer cell lines and in human cancer cell xenografts in mice (Campbell et al., 2004; Stefanska et al., 2013; Devailly et al., 2015). One way this could occur is MBD2 binding to hypermethylated promoters of tumor suppressor genes and contributing to their transcriptional silencing, which has been shown to occur in multiple human cancer cell lines (Lopez-Serra et al., 2008). Therefore, loss of MBD2 may be protective against tumorigenesis by relieving transcriptional repression of hypermethylated tumor suppressor genes such as *p14(ARF)* and *p16(INK4A)* that commonly show aberrant methylation in colon cancer cells (Magdinier and Wolffe, 2001; Martin et al., 2008). Similar mechanisms have been observed in glioma cells (Zhu et al., 2011) and breast cancer cells (Mian et al., 2011).

Conversely, downregulation of certain genes in the absence of MBD2 may be protective against tumorigenesis. MBD2 has been shown to directly repress human telomerase reverse transcriptase (*hTERT*) in several cancer cell types (Chatagnon et al., 2009). *hTERT* is usually hypermethylated and silenced, but is expressed in most cancer cells and therefore, in regards to this mechanism, loss of MBD2 may encourage tumor growth. In prostate cancer cells, loss of MBD2 suppresses tumor growth through hypermethylation and silencing of pro-metastatic genes (Shukeir et al., 2006). These complexities indicate that identification and manipulation of specific therapeutic pathways targeted via MBD2 will be challenging.

Studies of MBD2 in human cancer patients also point to MBD2 as a potential regulator of tumorigenesis. The finding that the 18q21 locus that includes *MBD2* is deleted in 70% of human colorectal cancers (Fearon et al., 1990) led to speculation that *MBD2* itself could be a candidate tumor suppressor gene. However, further investigation showed that only the deleted in colon cancer (*DCC*) gene at this locus is likely to be directly linked to cancer progression. All other neighboring genes, including *MBD2*, are rarely affected by hypermethylation or point mutations in colorectal cancer (Bader et al., 2003; Derks et al., 2009). *MBD3* is also generally unaffected by mutations or epigenetic changes in colon cancer (Zhu et al., 2004).

Despite the absence of mutations in *MBD2* or *MBD3* in cancer, there is evidence that these genes show altered regulation in tumors. Both MBD2 and MBD3 are downregulated in multiple human tumor types, but it is not clear what effect this has on tumor progression (Kanai et al., 1999; Müller-Tidow et al., 2001; Pontes et al., 2014). Studies of MBD2 in breast cancer have produced conflicting results. One study found that MBD2 is upregulated in breast tumors (Billard et al., 2002), while another found no difference (Müller et al., 2003). Analysis of single nucleotide polymorphisms in *MBD2* in breast cancer patients were similarly difficult to interpret, although some weak associations were detected (Zhu et al., 2005; Sapkota et al., 2014). Because MBD2 appears to have variable or context specific effects on tumorigenesis, significant further investigations into the molecular mechanisms of MBD2 function must be undertaken to identify potential therapeutic targets associated with these functions.

## Outstanding Questions on MBD Protein Function

An essential goal of the MBD protein field that remains unresolved is defining the specific functions of these proteins in a biologically relevant context. The MBD proteins are historically associated with transcriptional repression, yet attempts to identify specific methylated loci that are directly regulated and suppressed by these proteins *in vivo* do not fully support this model. Ablation of the MBD proteins, including MeCP2, MBD2, or MBD3 produces many subtle transcriptional changes without specific genes consistently showing upregulation, which would be expected according to models of MBD proteins as transcriptional repressors with direct targets (Chahrour et al., 2008; Günther et al., 2013). For MBD2, these questions are challenging to resolve because it is experimentally difficult to distinguish between functions that are MBD2-specific rather than mediated by the NuRD complex as a whole, while the role of MBD3 must also be considered. Numerous studies have relied on poorly characterized antibodies to MBD2, or antibodies that do not discriminate between MBD2 and MBD3. The recently described genetic tools consisting of mice expressing tagged MBD2 and MBD1 (Wood et al., 2016) will aid in future investigation of MBD protein functions *in vivo*.

One proposed explanation for why transcriptional repression is generally maintained in single MBD gene null mouse models is that the MBD family proteins are functionally redundant (Baubec and Schübeler, 2014). This hypothesis is most applicable to MBD2 and MBD3, which are very closely related both in their protein structure and as components of the NuRD complex (Hendrich and Bird, 1998; Le Guezennec et al., 2006). However, this model is undermined by the fact that each MBD protein has distinct loss-of-function phenotypes, spatiotemporal expression patterns, DNA binding properties, and protein complex interactions (Du et al., 2015; Wood et al., 2016). Therefore, the MBD proteins are unlikely to be functionally interchangeable *in vivo*.

Genetic evidence shows that loss of each MBD protein produces distinct phenotypes in mice (Guy et al., 2001; Hendrich et al., 2001; Allan et al., 2008). Loss of both MBD2 and MeCP2 in mice decreases survivability compared to loss of MeCP2 alone (Martín Caballero et al., 2009), while loss of MBD2 in *Mbd3* heterozygous mice resulted in decreased viability compared to *Mbd3* heterozygous mice with wild-type MBD2 expression (Hendrich et al., 2001). These findings indicate that MBD2 has other functions for which MeCP2 and MBD3 are unable to compensate. There is currently no information on the phenotype of mice with loss of both *Mbd1* and *Mbd2*, which would help clarify any potential interactions between these two MBD proteins. This has been difficult to achieve because the two genes are less than 4 Mb apart on the same chromosome.

MBD2 also shows a strikingly distinct spatiotemporal expression pattern at the protein level compared to MeCP2, MBD1, and MBD3 (**Figure 7**; Wood et al., 2016). It was shown that MBD2 has widespread expression, and is the only MBD protein of this group with detectable protein levels in multiple tissues. Therefore, MBD2 may have unrealized functions in these tissues, such as the spleen or small intestine, that may be linked to other reported functions for MBD2 in immunity and the gastrointestinal tract (Berger et al., 2007; Cook et al., 2015). Other molecular and biochemical evidence also suggest that MBD2 and MBD3 are not functionally interchangeable, despite their conservation and both being part of the NuRD complex (Le Guezennec et al., 2006). First, these proteins have entirely different DNA-binding capabilities, which is reflected in their distinct genome-wide binding profiles (Hashimoto et al., 2012a; Baubec et al., 2013; Günther et al., 2013). MBD2 and MBD3 also differ in their interactions with the NuRD complex, which could result in different context-specific roles (Le Guezennec et al., 2006; Baubec et al., 2013). Finally, MBD2 has numerous specific interactions with other protein complexes that influence how MBD2 interacts with NuRD (Angrisano et al., 2006; Tan and Nakielny, 2006). It is not clear if these other binding partners represent specific, NuRD-independent functions of MBD2, or if they serve to mediate the interactions between MBD2 and NuRD.

There are also many unanswered questions regarding the *in vivo* dynamics of the NuRD complex and MBD2 or MBD3. Biochemical evidence has shown that MBD2 and MBD3 are mutually exclusive in the NuRD complex and that the presence of the MBD protein is necessary for NuRD complex formation and function (Saito and Ishikawa, 2002; Le Guezennec et al., 2006; Ramírez et al., 2012). The spatiotemporal expression patterns of MBD2 and MBD3 raise further questions about NuRD formation and function, particularly in adult tissues where MBD3 is nearly undetectable but MBD2 is highly expressed (**Figure 7**; Wood et al., 2016). These findings may indicate that the NuRD complex necessarily forms with MBD2 in most peripheral adult tissues *in vivo* where MBD3 is absent or lowly expressed. Alternatively, the abundance of MBD2 over MBD3 may be indicative of other NuRD-independent MBD2 functions. With this model, it remains to be determined why loss of MBD3, even conditionally in specific tissues, has severe phenotypic consequences, while constitutive loss of MBD2 has only mild effects (Hendrich et al., 2001; Aguilera et al., 2011; Knock et al., 2015; Wood et al., 2016).

One possible model for MBD2, MBD3, and NuRD interactions is that MBD3 is essential for NuRD formation and function, while MBD2 represents a more transient member of NuRD that is required to fine-tune NuRD function and thus may be expendable. One study determined that most NuRD complexes in mammalian cells contain MBD3 rather than MBD2 (Zhang et al., 1999), but the dynamics of these interactions *in vivo* have not been fully determined. This scenario is supported by the distinct functions of MBD2 and MBD3 in ESCs. MBD3 is absolutely required for embryogenesis, while complete ablation of MBD2 does not compromise survivability or fertility (Hendrich et al., 2001). In contrast, different isoforms of MBD2 differentially regulate NuRD activity in ESCs (Lu et al., 2014). Genetic studies on MBD2 and MBD3 in the brain show a similar pattern, in which brain-specific loss of MBD3 is neonatal lethal, while loss of MBD2 does not severely impair brain functions (Knock et al., 2015; Wood et al., 2016). Additional biochemical evidence is necessary to determine if misregulation of NuRD activity occurs upon loss of MBD2 in the brain.

The different isoforms of MBD2 introduce further complications into models of MBD2/NuRD function. With a few important exceptions, most studies of MBD2 do not acknowledge or distinguish between the multiple alternatively spliced or translated isoforms. New evidence shows that experimentally distinguishing between the different isoforms of MBD2 may be essential to understanding this protein’s different functions *in vivo*. A recent study described opposing functions for MBD2a and MBD2c isoforms in the differentiation or proliferation of ESCs (Lu et al., 2014). Biochemical evidence shows that MBD2a, MBD2b, and MBD2c have different interactions with NuRD and other protein complexes and are recruited to DNA differently (Tan and Nakielny, 2006; Baubec et al., 2013; Lu et al., 2014). These multiple isoforms of MBD2 may differentially regulate NuRD activity in other cell types besides ESCs through mechanisms that have yet to be fully explored. Therefore, while complete deletion of MBD2 does not have severe phenotypic effects, it is possible that disrupting the balance of MBD2 isoform expression could be more detrimental to NuRD regulation and therefore produce more robust phenotypes.

In summary, MBD2 is an integral part of the NuRD complex with many unanswered questions regarding its molecular and biological functions. In order to address these questions, it is essential to unravel the complexities of different isoforms of MBD2 in association with NuRD. The three isoforms of MBD2 each have different interactions with NuRD and other protein complexes, which may contribute to their distinct functions. In addition, MBD2 activity must be considered in the context of MBD3 and NuRD, especially *in vivo* where MBD2 and MBD3 have distinct spatiotemporal expression patterns. These complex isoforms and interactions may underlie the surprisingly mild phenotype of *Mbd2* knockout mice. Future investigations into MBD2 functions may have important implications for the study of pluripotency, immunity, and cancer, in addition to revealing insights into broader epigenetic mechanisms. The recent development of tagged MBD2 knockin mice provides a powerful tool to address these questions through future *in vivo* mechanistic studies (Wood et al., 2016).

## Author Contributions

All authors listed have made substantial, direct and intellectual contribution to the work, and approved it for publication.

## Conflict of Interest Statement

The authors declare that the research was conducted in the absence of any commercial or financial relationships that could be construed as a potential conflict of interest.
